# Meningococcal Carriage in ‘Men Having Sex With Men’ With Pharyngeal Gonorrhoea

**DOI:** 10.3389/fcimb.2021.798575

**Published:** 2022-01-12

**Authors:** Sara Morselli, Valeria Gaspari, Alessia Cantiani, Melissa Salvo, Claudio Foschi, Tiziana Lazzarotto, Antonella Marangoni

**Affiliations:** ^1^ Microbiology, Department of Specialized, Experimental and Diagnostic Medicine (DIMES), University of Bologna, Bologna, Italy; ^2^ Dermatology Unit, IRCCS S. Orsola-Malpighi Hospital, Bologna, Italy

**Keywords:** *Neisseria meningitidis*, *Neisseria gonorrhoeae*, oro-pharynx, MSM, meningococcal carriage

## Abstract

We assessed the characteristics of *Neisseria meningitidis* pharyngeal carriage in a cohort of ‘men having sex with men’, including patients with pharyngeal *Neisseria gonorrhoeae* infection. In the period 2017-2019, among all the oropharyngeal samples tested for gonorrhoea from MSM attending a STI Clinic in Bologna (Italy), we randomly selected 244 *N. gonorrhoeae*-positive samples and 403 negatives (n=647). Pharyngeal specimens were tested for *N. meningitidis* presence, by the detection of *sodC* gene. *N. meningitidis*-positive samples were further grouped by PCR tests for the major invasive genogroups (i.e., A, B, C, W, and Y). A molecular assay, targeting capsule transporter gene, was used to determine meningococcal capsular status. Overall, 75.8% (491/647) of samples tested positive for *sodC* gene, indicating a pharyngeal meningococcal carriage. Meningococcal colonisation was significantly more frequent in younger subjects (*P*=0.009), with no association with HIV infection. Non-groupable meningococci represented most of pharyngeal carriages (about 71%). The commonest *N. meningitidis* serogroup was B (23.6%), followed by C (2.1%), Y (1.8%) and W (1.1%). Meningococci were often characterized by the genetic potential of capsule production. Interestingly, a negative association between *N. meningitidis* and *N. gonorrhoeae* was found: pharyngeal gonorrhoea was significantly more present in patients without meningococcal carriage (*P*=0.03). Although preliminary, our data added knowledge on the epidemiology of meningococcal carriage in MSM communities at high risk of gonococcal infections, gaining new insights into the interactions/dynamics between *N. meningitidis* and *N. gonorrhoeae*.

## Introduction

*Neisseria meningitidis* (NM) and *Neisseria gonorrhoeae* (GC) are two closely related Gram-negative coccoid bacteria (80-90% genetic homology), expressing different pathogenicity and responsible of different clinical conditions (Tinsley and Nassif, 1996).

NM is usually a commensal microorganism, able to colonize the nasopharyngeal niche without affecting the host (Gentile et al., 2021). Meningococcal asymptomatic carriage can be transient, being more common in adolescent and young adults. Occasionally, NM can invade normally sterile sites (e.g., bloodstream, cerebrospinal fluid), causing an invasive meningococcal disease, characterized by meningitis and/or sepsis (Igidbashian et al., 2020). Disease progress is usually acute and severe, with a high mortality rate (10%-15%) and a significant risk of long-term sequelae (60%) (CDC, 2015).

On the other side, GC is the causative agent of gonorrhoea, one of the most common bacterial sexually transmitted infections (STIs) worldwide, with a significant clinical and public health impact (ECDC, 2020). Indeed, gonococcal genital infections (e.g., urethritis and cervicitis) can be associated with serious complications such as pelvic inflammatory disease, infertility, and ectopic pregnancy (Morgan and Decker, 2016).

Besides the common urogenital localizations, GC infections can be found at extra-genital sites, such as pharyngeal mucosa, mainly in ‘men who have sex with men’ (MSM) reporting unsafe oral intercourse (Gaspari et al., 2019). Pharyngeal infections are usually characterized by the absence of symptoms, acting as an important reservoir for their further spread (Gaspari et al., 2019).

From what has been said so far, it is evident that both NM and GC can be found in the oro-pharynx, that represents a suitable ecological niche for *Neisseria* species to replicate and persist over time (Marangoni et al., 2020a).

The oro-pharynx is also a crucial site for the emergence of multi-drug resistance in GC. Indeed, the mosaic *penA* alleles, responsible for cephalosporin resistance, have emerged by DNA recombination with partial *penA* genes, particularly belonging to commensal pharyngeal *Neisseria* species, including *Neisseria meningitidis* (Marangoni et al., 2020b).

Moreover, previous studies, focusing on the interactions between commensals and pathogenic *Neisseria* species of the oropharynx, demonstrated that commensals can compete with pathogens and, thus, provide protection from colonization and invasion. For example, *N. lactamica* can displace *N. meningitidis* from the nasopharynx and hinder meningococcal acquisition (Deasy et al., 2015).

The importance of deepening the relationships between NM and GC is strengthened by the idea that immunity to gonorrhoea can be achieved in humans through vaccination with the serogroup B NM outer membrane vesicle (OMV) vaccine (Paynter et al., 2019; Leduc et al., 2020). Indeed, epidemiological evidence from Cuba, Brazil, and New Zealand clearly shows a marked decline in gonorrhoea incidence following implementation of the meningococcal group B OMV vaccines (Petousis-Harris, 2018).

Therefore, in this study we aimed to evaluate the possible correlations between the presence of NM and GC in the oropharyngeal niche, focusing on a high-risk group. Considering that MSM show high rates of pharyngeal gonorrhoea and are, contemporary, at increased risk for invasive meningococcal disease (Folaranmi et al., 2017; Gaspari et al., 2019), this population is particularly suitable to study NM-GC relationships.

In particular, we assessed the characteristics of meningococcal carriage in a cohort of MSM, including two different groups of patients, i.e., subjects with pharyngeal gonorrhoea and GC-negative subjects. The following investigations were performed: (i) research of meningococcal carriage rates, (ii) evaluation of the presence and distribution of major NM invasive serogroups (i.e., A, B, C, Y, W), (iii) assessment of NM potential ability of capsule synthesis and transport.

## Materials and Methods

### Study Population and Sample Collection

In the period 2017-2019, among all the oropharyngeal samples tested for gonorrhoea from MSM attending the STI Outpatients Clinic of St. Orsola Malpighi Hospital in Bologna (Italy), we randomly selected 244 *N. gonorrhoeae*-positive samples and 403 negatives (n=647).

Samples (E-Swab, Copan, Brescia, Italy) were collected during the routine screening for extra-genital STIs, strongly recommended for patients reporting history of unsafe intercourse.

GC positive and negative samples were matched for age and collected when no invasive meningococcal outbreak was present. The distribution of samples per year was as follows: 111 samples collected in 2017, of which 15.3% GC-positive; 281 samples collected in 2018, of which, 37% GC-positive; 255 samples collected in 2019, of which 51.7% GC-positive.

Diagnosis of GC infection was based on a commercial duplex real-time PCR test simultaneously detecting the presence of *C. trachomatis* and *N. gonorrhoeae* DNA (Versant CT/GC DNA 1.0 Assay (Siemens Healthcare Diagnostics, Tarrytown, NY, USA) (Marangoni et al., 2015).

Samples collected during the follow-up period of GC-positive patients, as well as multiple samples collected from the same patient, were excluded from the study.

Personal information (e.g., age), and data about HIV serostatus were recorded from each patient.

The Ethical Committee of the Hospital approved the study protocol (78/2017/U/Tess), and all subjects gave written informed consent to the work.

### Detection, Typing, and Capsular Status of *Neisseria meningitidis*

Starting from the remaining DNA eluate of the Versant PCR plate, all the pharyngeal swabs were tested for the presence of NM, by means of the detection of *sodC* gene, encoding Cu-Zn superoxide dismutase, as previously described (Dolan Thomas et al., 2011). This molecular assay proved to be highly sensitive and specific for NM detection, with no false-positive results due to non-meningococcal *Neisseria* species (Dolan Thomas et al., 2011). According to the authors, cycle threshold (Ct) values ≤35 were considered positive, Ct values in the range of 36–40 equivocal, and Ct values >40 negative. Equivocal specimens were diluted 1∶4 and 1∶10 and re-tested in duplicate, in order to reduce possible inhibitors. If the average Ct of the diluted specimen fell below 35, that specimen was considered positive, whereas if the average Ct of the diluted specimen remained in the 35–40 range, that specimen was considered negative.

NM-positive samples were subsequently grouped (i.e., detection of the capsular polysaccharide genes) by real-time PCR assays, targeting the five genogroups responsible for greater than 90% of the invasive disease worldwide (i.e., A, B, C, W, and Y) (**Table 1**) (McHugh et al., 2015; Rojas et al., 2015; Clark et al., 2019)

**Table 1:** Real-time PCR primers and probes used for *N. meningitidis* detection, typing, and assessment of capsular status.

Primers/probes	Sequence (5’ - 3’)	Target
Nm *sodC* forward Nm *sodC* reverse Nm *sodC* probe	GCACACTTAGGTGATTTACCTGCAT CCACCCGTGTGGATCATAATAGA (FAM)-CATGATGGCACAGCAACAAATCCTGTT T	Detection of NM (*sodC* gene)
Nm csa forward Nm csa reverse Nm csa probe	GCCACAAAGTGCCCTTCCT TGGTATATGGTGCAAGCTGGTT (FAM)-TTTAGCTCACATGCTATTG	NM typing (group A)
Nm csb forward Nm csb reverse Nm csb probe	ATTATACAGCCTGCTCATCTCTATATGC TCCCTTCATCAATTAAATGAGTCGTA (FAM)-TTACAGGCCACTACTCCT	NM typing (group B)
Nm csc forward Nm csc reverse Nm csc probe	GCACATTCAGGCGGGATTA TTGAGATATGCGGTATTTGTCTTGA (FAM)-ACAAGCCAATCTATTGCT	NM typing (group C)
Nm csy forward Nm csy reverse Nm csy probe	GTACGATATCCCTATCCTTGCCTATAA CCATTCCAGAAATATCACCAGTTTTA (FAM)-TGGAGCGAATGATTTTAGCAA	NM typing (group Y)
Nm csw forward Nm csw reverse Nm csw probe	TGGTGTATGATATTCCAATCGTTGT CCATTCCAGAAATATCACCAGTTTT (FAM)-AGCGAATGATTACAGTAACT	NM typing (group W)
Nm *ctrA* forward Nm *ctrA* reverse 1 Nm *ctrA* reverse 2 Nm *ctrA* probe	GCTGCGGTAGGTGGTTCAA TTGTCGCGGATTTGCAACTA TTGCCGCGGATTGGCCACCA (FAM)-CATTGCCACGTGTCAGCTGCACAT	Capsule transporter gene (*ctrA*)

Finally, to determine the capsular status of the carried meningococci, a further real-time PCR assay for NM capsule transporter gene (*ctrA*) was used, as well (McHugh et al., 2015).

Primers and hydrolysis probes for all the molecular assays used in this study are reported in **Table 1**.

The PCR reaction mixtures (final volume: 25 μL) included 12.5 μL of Platinum Quantitative PCR Supermix-UDG with ROX (Invitrogen), 300 nM of primers, 100 nM of the probe, 5 mM of MgCl_2_, and 2.5 μL of template. All PCR reactions were performed with the following cycling conditions using a QuantStudio Real-Time PCR system (Applied Biosystems, Japan): 2 min at 50°C, 10 min at 95°C, and 40 cycles of 15 s at 95°C and 60 s at 60°C.

### Statistical Analysis

Differences in clinical and demographic parameters were tested by Fisher’s exact test for categorical data and t-test for quantitative data, using Prism 5.02 version for Windows (GraphPad Software, San Diego California USA, www.graphpad.com). A *P* value < 0.05 was considered as statistically significant.

## Results

### Study Population and Samples

The mean age of MSM whose pharyngeal swabs were collected and analysed was 33.1 ± 9.8 years (min-max: 19-77 years). No significant difference was observed between the mean age of GC-positive vs negative subjects (32.6 ± 9.3 vs 33.5 ± 10.1 years; *P*=0.28). Overall, 126 samples (19.4%) belonged to HIV-positive patients. HIV-positive MSM were significantly older than HIV-negative ones (38.4 ± 9.7 vs 31.9 ± 9.4; *P*<0.001). No association between HIV-positivity and GC pharyngeal infection was found (*P*=0.26).

### Prevalence of Meningococcal Carriage and NM Typing

Among the 647 specimens collected for the study, 491 samples (491/647; 75.8%) tested positive for the presence of *sodC* gene, indicating a pharyngeal meningococcal carriage.

**Figure 1:**
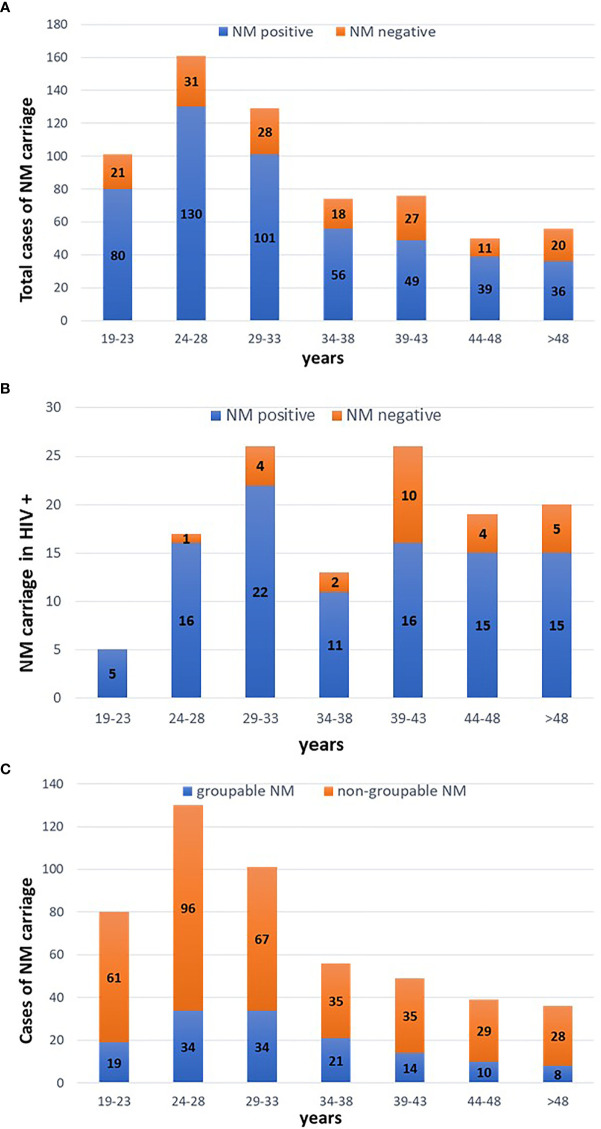
Cases of meningococcal carriage stratified by age. Five-years periods were considered. **(A)** distribution of NM positive and negative cases considering all the MSM included in the study. **(B)** distribution of NM positive and negative cases in HIV positive people. **(C)** distribution of groupable and non-groupable NM cases.

Samples positive for NM carriage peaked among subjects aged between 24-28 years (130 cases; 80% of MSM enrolled in this age range). A decrease of NM colonisation was observed with the increasing age (**Figure 1**, panel A). Considering only HIV positive people, the prevalence of NM carriers was higher in subjects aged between 19-23 (100%) and 24-28 (94%) years (**Figure 1**, panel B).

**Figure 2:**
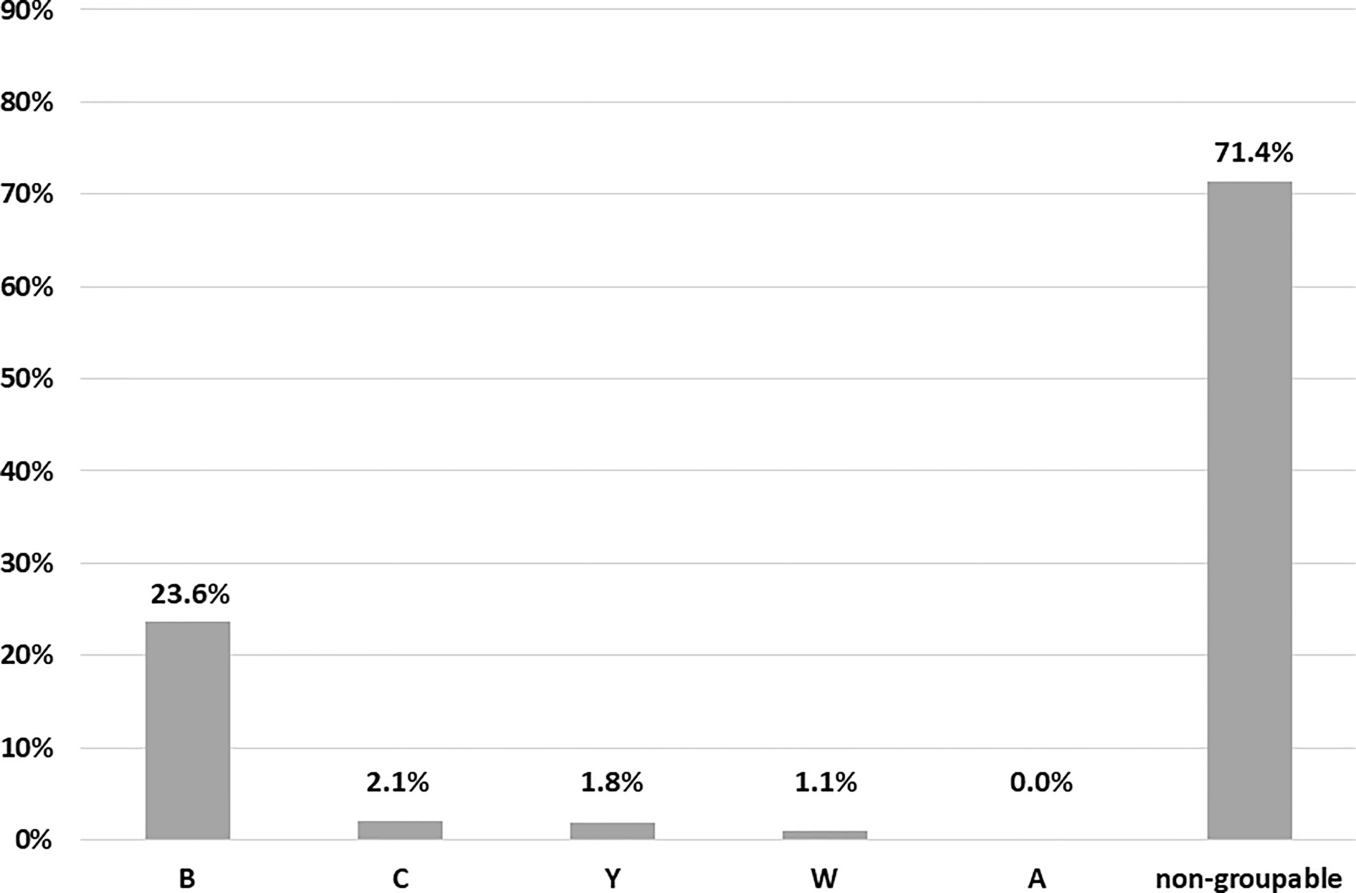
Characteristics of meningococcal pharyngeal carriage. Distribution of NM serogroups (the five major invasive serogroups were searched, namely A, B, C, Y, W).

Serogroup B accounted for most of NM-positive cases (116/491; 23.6%), followed by serogroup C (10 cases; 2.1%), Y (9 cases; 1.8%) and W (5 cases; 1.1%). No cases of serogroup A were detected. All the remaining samples (71.4%) yielded a negative result when tested for the five major NM genogroups, being defined as ‘non-groupable’ (**Figure 2**).

Overall, the prevalence of potentially ‘major invasive’ NM serogroups (i.e., B, C, Y, W) was 21.6% (140/647); these serogroups represented the 28.5% (140/491) of all cases of meningococcal carriage. In HIV positive people, one third of NM cases (31%; 31/100) was due to groupable meningococci, mainly B (28/31).

As shown in **Figure 1** (panel C), prevalence of groupable NM cases was higher in MSM aged between 34-38 (37.5%) and 29-33 years (33.6%).

Most of genotyped NM cases were characterized by the positivity of capsule transporter gene (*ctrA*), indicating the genetic potential of capsule production (96.6% of serogroup B, 80% of serogroups C and W, 78% of serogroup Y).

### Association Between NM, GC, and Specific Variables

Interestingly, meningococcal carriage was significantly associated with the absence of pharyngeal gonorrhoea. Indeed, GC was found in 35.4% of NM-positive patients, compared to 44.8% of NM-negative subjects (*P*=0.03) (**Table 2**).

**Table 2:** Characteristics of meningococcal carriage.

	NM positive (n = 491)	NM negative (n = 156)	*P* value
**GC positivity (n=244)**	35.4% (174/491)	44.8% (70/156)	0.03*
**HIV positivity (n=126)**	20.3% (100/491)	16.6% (26/156)	0.35
**CT positivity (n=47)**	6.7% (33/491)	8.9% (14/156)	0.37
**Age (years; mean ± SD)**	32.6 ± 9.7	34.9 ± 10.0	0.009*

*statistically significant.The table shows the associations between NM pharyngeal carriage and available variables. Fisher’s exact test was used to assess statistical significance for categorical data (i.e., GC, HIV and CT positivity), whereas t-test for quantitative data (age).

No significant associations were found between GC presence and NM type (‘groupable’ vs non-groupable meningococci; serogroup B vs other NM types) (data not shown).

Pharyngeal NM carriage was not correlated with HIV positivity (*P*=0.35) nor with pharyngeal *C. trachomatis* infection (*P*=0.37) (**Table 2**).

Finally, it is worth mentioning that the presence of NM was positively associated with the younger age of patients (*P*=0.009) (**Table 2**).

## Discussion

The recent demonstration of the efficacy of the meningococcal vaccine against gonorrhoea gave new life to studies about NM-GC interaction, with the aim of implementing new strategies for the prevention of both infections, mainly in high-risk groups (Petousis-Harris, 2018).

In this context, the goal of this study was to evaluate the characteristics of meningococcal pharyngeal carriage in subjects attending a STIs Clinic for gonorrhoea screening.

We focused on a cohort of MSM, considering that, in this population, GC sexually transmitted pharyngeal infections are very common, and, at the same time, there is an increased risk for meningococcal invasive disease, in conjunction with HIV infection (Folaranmi et al., 2017; Gaspari et al., 2019).

A total of 647 pharyngeal swabs collected from MSM, including 244 GC-positive, were analysed for the presence of a meningococcal carriage, by means of nucleic acid amplification techniques (NAAT). NM-positive samples were further examined to identify the most common meningococcal serogroups (i.e., A, B, C, Y, W) and to assess the genetic potential of capsular expression.

At first, we observed a rather high prevalence of meningococcal pharyngeal colonization among the enrolled MSM, exceeding the 70%. The rate of meningococcal carriage found in our study was much higher compared to previous studies performed among MSM groups, showing a prevalence ranging between 25% and 30% (Ngai et al., 2020; Tinggaard et al., 2021). However, considering only cases belonging to the most common ‘invasive’ genogroups, the prevalence of NM-positive cases dropped to 21%.

A previous investigation on meningococcal pharyngeal presence stratified for sexual habits showed that homosexuals had the highest carriage (23.8%) and heterosexual females the lowest (5.9%), with significant differences in carriage rates between homosexual and heterosexual men (Russell et al., 1995).

The surprisingly high rate of meningococcal carriage found in our cohort could be due to the fact that our MSM population represents a community living in the same high density urban area, with frequent closed-contacts and sharing common recreational activities.

It is worth mentioning that, in ‘closed’ communities, carriage rates may be significantly higher and that same-sex intercourses represent a significant risk factor for meningococcal pharyngeal colonisation (Miglietta et al., 2019; Gentile et al., 2021).

Moreover, considering that NM detection was based solely on PCR assays, we cannot rule out that part of the positive results is due to residual nucleic acids from non-viable microorganisms. Thus, future studies based on culture techniques will help elucidating the exact prevalence of NM carriage in the MSM population in our area.

In addition, we found that meningococcal carriage was significantly more frequent in younger subjects. These data are in line with previous reports, showing that younger groups reach the highest rates of meningococcal carriage. Indeed, in Europe, pharyngeal NM carriage shows a peak during the adolescence and young adulthood (approximately 24%), being significantly lower (<10%) in adults over 50 years of age (Christensen et al., 2010). Moreover, it should be noted that meningococcal colonisation is also influenced by social factors typical of younger people, including going to clubs, kissing, smoking, and living in closed environments such as university dormitories (Bidmos et al., 2011; Tekin et al., 2017).

Other interesting data emerged from NM typing (i.e., detection of the most common invasive serogroups) and the assessment of the genetic potential to express various capsule groups. In agreement with previous findings, non-groupable meningococci (referred to meningococci other than A, B, C, W-135 and Y) represented most of pharyngeal carriages (about 70%) (Tekin et al., 2017; Miglietta et al., 2019). Beyond these cases, the most common NM serogroup was B, followed by C, Y, and W. This distribution is similar to the one reported from previous national and European surveys, showing the predominancy of serogroup B, with the absence of serogroup A (ECDC, 2019; Peterson et al., 2019).

Most of genotyped NM cases were characterized by the potential ability of capsule synthesis and transport (i.e., positivity of capsule transporter gene, *ctrA*). As previously described, in agreement with our results, the capsule locus, including *ctrA* gene, is subject to rearrangement, and 16% or more of carried meningococci have been shown to lack *ctrA* altogether (Dolan Thomas et al., 2011).

Secondly, we investigated the association between GC and NM presence in the pharyngeal environment. Interestingly, a negative correlation between these two microbial species was found: indeed, pharyngeal gonorrhoea was significantly more present in patients with no meningococcal carriage.

Even though the cause-and-effect relationship between NM carriage and GC positivity could not be clarified with our data, we can hypothesize that the presence of meningococci in the pharyngeal niche could represent a potential protective factor for the acquisition and transmission of gonorrhoea. It is also possible that pharyngeal GC acquisition prevented the subject from meningococcal carriage.

It has been recently demonstrated that other commensal *Neisseria* species, such as *N. lactamica* can kill GC through a mechanism based on genetic competence and DNA methylation state (Kim et al., 2019). Therefore, we cannot exclude with certainty an additional role of other *Neisseria* species in the dynamic interaction between GC and meningococci.

Commensal bacteria are efficient promoters of mucosal lymphoid tissue development and *Neisseria* species establish themselves in the mucosa where IgA and transduction of IgG are the main humoral effectors. In this regard, it has been shown that human parotid saliva positive for NM IgA has a cross-recognition against GC (Brandtzaeg, 2007).

In this context, previous studies suggest that vaccination with the meningococcal B OMV vaccine can significantly reduce gonorrhoea infection rates, indicating a possible cross-species protection against GC (Petousis-Harris, 2018; Paynter et al., 2019; Leduc et al., 2020).

Nevertheless, in contrast with these data, it has been recently shown that urethral infection with ‘NM urethritis clade’ does not protect against gonorrhea despite substantial sequence similarities in shared protein antigens (Norris Turner et al., 2021).

In conclusion, we investigated the association between the presence of NM colonisation and gonorrhoea infection in the oro-pharynx, strengthening the importance of a possible role of immunity in meningococcal carriers. Although preliminary, these data can help gain new insights into NM-GC interactions/dynamics, in order to better understand the pathobiology of these microorganisms, as well as to set new strategies for the prevention of gonorrhoea. In absence of a gonococcal vaccine and in view of the growing problem of GC antimicrobial resistance, there may be a role for meningococcal vaccines in programmes targeting adolescents and groups at high risk for both meningococcal disease and gonorrhoea (Petousis-Harris, 2018).

In Italy, meningococcal vaccines (both MenACWY and MenB vaccines) are routinely recommended in people living with HIV, especially in presence of specific risk factors (Sticchi et al., 2017). Data about vaccine adherence in HIV-positive patients are limited, with no exact information about meningococcal vaccine coverage (De Macina et al., 2018). Conversely, it has been estimated that conjugate vaccine (MenACWY) coverage in adolescent in 2020 exceeds the 55%, with a significant variability between Italian regions (available at: https://www.salute.gov.it/portale/documentazione/p6_2_8_3_1.jsp?lingua=italiano&id=20). Thus, future health policy programmes should assess the adherence, the impact, and the cost-effectiveness ratio of meningococcal vaccines in specific subgroups of subjects.

We are fully aware of some limitations of this study. At first, this is a single centre study of MSM of one STI Clinic, and our findings should be validated in larger cohorts of subjects. Second, we assessed only the presence of the five major NM serogroups (A, B, C, W-135 and Y), making it necessary to include additional PCR tests for the less frequent invasive meningococcal types (e.g., E, X, Z) (Tzeng and Stephens, 2021). This will help to truly assess the proportion of ‘non-groupable’ samples in our cohort. Finally, the non-homogeneous collection of NG positive samples during the study period limited the possibility of more in-depth analyses (e.g., assessment of the distribution of meningococcal carriage between winter and summer periods).

Future perspectives will include: (i) the use of culture-based approaches to better understand the prevalence and characteristics of NM carriage, including the ability of meningococcal capsular production, (ii) the collection of information about meningococcal vaccination status of the subject enrolled.

## Data Availability Statement

The raw data supporting the conclusions of this article will be made available by the authors, without undue reservation.

## Ethics Statement

The study protocol was reviewed and approved by the Ethical Committee of S.Orsola-Malpighi Hospital (78/2017/U/Tess). The patients/participants provided their written informed consent to participate in this study.

## Author Contributions

AM and CF conceived and designed the study. VG collected the samples. CF, MS, SM, and AC performed the experiments. CF, SM, and MS analysed the data. AM and TL contributed reagents/materials/analysis tools. AM, CF, and SM wrote the paper. All authors read, reviewed, and approved the final manuscript.

## Funding

This research received no specific grant from any funding agency in the public, commercial, or not-for-profit sectors.

## Conflict of Interest

The authors declare that the research was conducted in the absence of any commercial or financial relationships that could be construed as a potential conflict of interest.

## Publisher’s Note

All claims expressed in this article are solely those of the authors and do not necessarily represent those of their affiliated organizations, or those of the publisher, the editors and the reviewers. Any product that may be evaluated in this article, or claim that may be made by its manufacturer, is not guaranteed or endorsed by the publisher.
